# Tunable quantum interference in a 3D integrated circuit

**DOI:** 10.1038/srep09601

**Published:** 2015-04-27

**Authors:** Zachary Chaboyer, Thomas Meany, L. G. Helt, Michael J. Withford, M. J. Steel

**Affiliations:** 1 Centre for Ultrahigh bandwidth Devices for Optical Systems (CUDOS), MQ Photonics Research Centre, Department of Physics and Astronomy, Macquarie University, NSW 2109, Australia

## Abstract

Integrated photonics promises solutions to questions of stability, complexity, and size in quantum optics. Advances in tunable and non-planar integrated platforms, such as laser-inscribed photonics, continue to bring the realisation of quantum advantages in computation and metrology ever closer, perhaps most easily seen in multi-path interferometry. Here we demonstrate control of two-photon interference in a chip-scale 3D multi-path interferometer, showing a reduced periodicity and enhanced visibility compared to single photon measurements. Observed non-classical visibilities are widely tunable, and explained well by theoretical predictions based on classical measurements. With these predictions we extract Fisher information approaching a theoretical maximum. Our results open a path to quantum enhanced phase measurements.

When an *N* photon Fock state passes through an optical delay it behaves as a single object with a momentum *N* times larger than that of the corresponding single photon state^1^. For path-entangled photons in the two arms of a Mach-Zehnder interferometer, either *N* in the upper arm and 0 in the lower, or 0 in the upper arm and *N* in the lower (so called “N00N” states), this phenomenon leads to a reduced peak to peak distance in interference fringes seen at the output, enabling quantum-enhanced phase estimation^2^,^3^,^4^ and, in theory, higher-resolution lithography^5^. More recently, attention has been devoted to further improving the precision of quantum interferometric schemes by employing optimized input states^6^,^7^ and adaptive feedback phases^8^,^9^, as well as developing schemes that are robust to photon loss^10^. However, the majority of these efforts have employed conventional two-path Mach-Zehnder and Michelson interferometers.

Additional gains can be realised by increasing the number of paths available for photons to take in an interferometer^11^, including protocols for multiparameter estimation^12^,^13^ and a reduced sensitivity to photon loss^14^. However, limitations of existing technology have to this point prevented experimental demonstrations of non-classical interference in multi-path interferometers. Although such devices can be implemented relatively straightforwardly using single-mode fibre components^15^, utilizing these fibre devices for quantum interferometry is made difficult by the phase instability caused by thermal and acoustic noise^16^. For this reason, experimental quantum interferometry in multi-arm devices has remained largely unexplored despite its potential advantages.

Here we report on the quantum enhancement in fringe periodicity observed when passing pairs of photons through two integrated three-port splitters placed in series to form a three-path analogue of a Mach-Zehnder interferometer^17^ (see Fig. 1). Multiphoton interference at the first three-way splitter, or “tritter”, causes the photons to coalesce into a superposition of photons occupying each interferometer arm^18^,^19^,^20^. The photons then probabailistically pass through the same phase in the measurement arm, resulting in a known subset of two-photon fringes exhibiting both a reduced periodicity and higher visibility than would be obtained classically. By mounting a thermo-optic phase shifter onto the chip, we are able to adjust the relative phase in one of the interferometer arms. This tunability allows us to control the visibility of the two-photon quantum interference that occurs within the device, thereby allowing for a measurement of two-photon fringes with enhanced periodicity. We close with a discussion on the applicability of our device to quantum-enhanced phase measurement.

## Results

### Device fabrication and illumination

**Figure 1:**
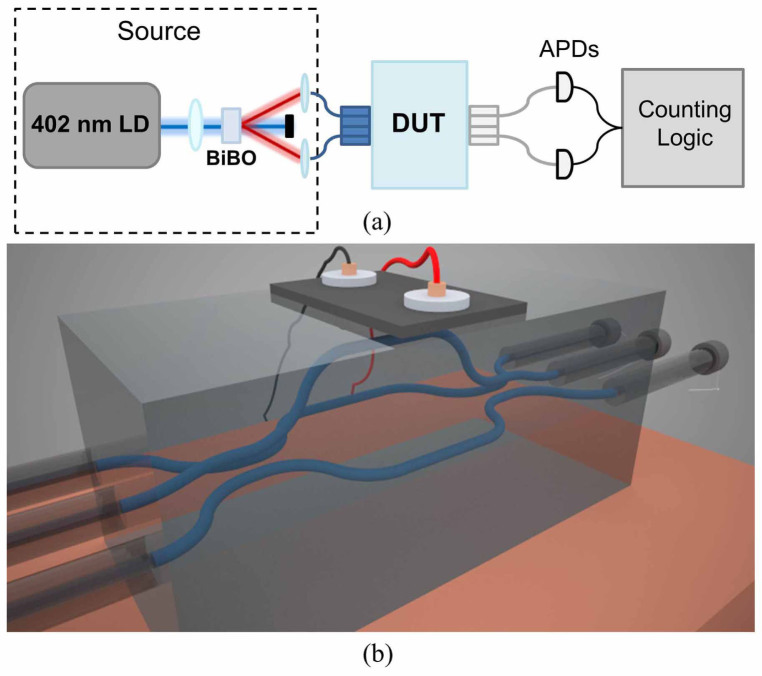
(a) Schematic of the experiment. LD: laser diode, BiBO: bismuth borate crystal, DUT: device under test, APDs: avalanche photo diodes. (b) Representation of the 3D interferometer, the direct laser inscription method employed here enables the single step fabrication of a truly 3D circuit. The middle interferometer arm is raised with respect to the others, imparting a relative phase due to interaction with a heater placed on the chip surface.

While precise control of non-classical interference has been demonstrated in two-arm Mach-Zehnder interferometers fabricated using planar silica-on-silicon^21^ and UV-writing technologies^22^, these platforms require multiports to be implemented as multimode interference devices^23^, which have low fabrication tolerances, or as complex concatenations of 50/50 beamsplitters and phase shifters^24^. Therefore, we employed the femtosecond laser direct-write (FLDW) technique^25^ to enable stable multi-arm quantum interferometry in a 40 mm long alumino-borosilicate glass chip (see Methods). The middle interferometer arm was raised 127 *μ*m above the other two arms to allow for interaction with a heating element mounted on the chip surface that acts as a thermo-optic phase shifter (see Fig. 1).

The fabricated three-arm interferometer was excited using photons from a type I spontaneous parametric down-conversion (SPDC) source that produced degenerate photons at 804 nm. The photons were coupled in and out of the chip using optical fibres aligned in V-groove arrays. Single photon detection was performed using silicon avalanche photodiodes (see Methods for details).

### Circuit characteristics

Propagation losses of approximately 0.3 dB/cm have been previously measured^26^,^27^ for waveguides that are fabricated identically to those making up our interferometer. By comparison, the total insertion losses are up to an order of magnitude higher (see methods). Therefore, in characterising the device, we assume that the majority of losses occur outside the chip, allowing the interferometer to be well approximated mathematically by a unitary operation 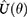. This unitary operation is modified by applying a voltage across the heater mounted on the surface, leading to a change in the temperature of the top surface that is proportional to the power dissipated by the heater. Since the heater is large compared with the lateral dimensions of the waveguide device, the temperature gradient set up inside the chip within the vicinity of the interferometer arms may be described by a simple 1D heat flow model. When the bottom surface is held at a constant temperature the system approaches a linear gradient in the steady state so that the temperature of the raised waveguide is increased with respect to the other two (see supplementary material). This leads to an overall phase shift in the middle interferometer arm represented by the quantity *θ*.

**Figure 2:**
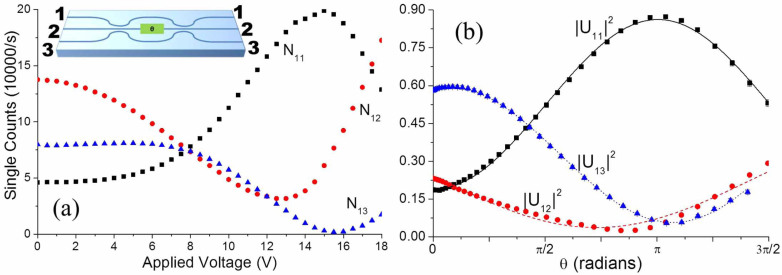
(a) Single photon fringes measured when launching into port 1. Poisson error bars are smaller than the data points and are omitted. Inset: schematic of the device showing the numbering convention for the input and output ports. (b) |*U*_1*j*_|^2^ extracted from the raw single photon data plotted as a function of induced phase. Black squares: |*U*_11_|^2^, red circles: |*U*_12_|^2^, blue triangles: |*U*_13_|^2^, curves: fits to |*U*_1*j*_(*θ*)|^2^ = *A* sin*θ + B* cos*θ*.

A state 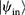 injected into the device evolves into the state 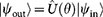, and in the photon spatial mode basis representation the unitary operator takes the form of a 3 × 3 matrix connecting the input modes 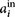 to the output modes 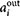 (see supplementary information):
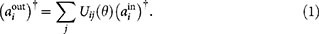
The nonlinear relationship between the induced phase *θ* and the applied voltage *V* was established by single-photon characterisation of the nine possible classical interference patterns, examining the count rates from each output port for separate excitation of each input port. Performing single-photon measurements using the same SPDC source allowed us to ensure consistency between our classical characterisation and the subsequent non-classical study. The measured counts as a function of voltage when injecting into input port 1 are shown in Fig. 2(a). Since the input and output coupling losses at each port 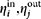 are unknown, the unitary matrix elements *U_ij_*(*θ*(*V*)) at each voltage were determined by maximum-likelihood estimation applied to ratios of the measured count rates that are independent of the facet losses^19^ (see supplementary material). To first approximation, the thermo-optic phase change depends linearly on the dissipated power as *θ* = *kIV*, where *I* is the current in the heater at given voltage *V*. The matrix elements *U_ij_*(*θ*) contain phase terms of the form *e^iθ^*, and their squared moduli were fitted to a function taking the form |*U_ij_*(*kIV*)|^2^ = *A* sin(*kIV*) + *B* cos(*kIV*). After normalisation and fitting, we determined a proportionality constant of *k* = 0.626 ± 0.031 W^−1^. The normalised and fitted classical fringes for input 1 are shown in Fig. 2(b) (see supplementary material for others).

### Quantum Characterisation

**Figure 3:**
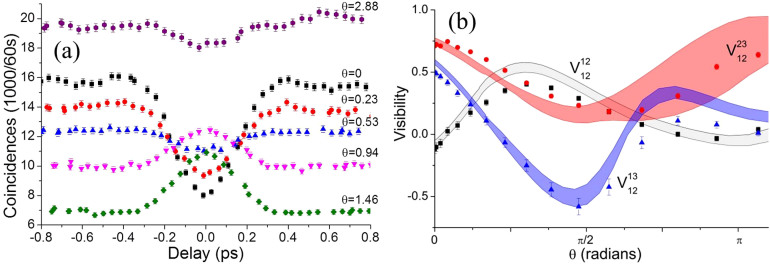
(a) Two-photon coincidences as a function of relative delay at various values of phase *θ* injecting into inputs 1 and 2 and measuring at outputs 1 and 3. (b) Measured (points) and predicted (bands) two-photon visibilities as a function of *θ* when injecting |110〉. Black squares: 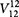, red circles: 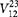, blue triangles: 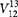. Grey band: predicted upper and lower bounds for 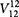, red band: predicted bounds for 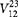, blue band: predicted bounds for 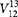.

We then proceeded to the quantum characterisation of the tunable device by injecting photon pairs into each combination of input ports, corresponding to the Fock states 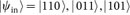. The distinguishability of the photons was controlled by adjusting their relative arrival time at the chip by means of a free space delay. Scans of the delay 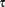 were performed while varying *θ* and measuring coincidence counts between photons emerging from each combination of output ports. The change in the output state 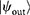 with *θ* was monitored as a change in the degree of quantum interference in a two-photon experiment as quantified by the visibility
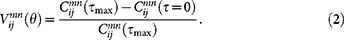
Here 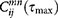 is the measured coincidences at outputs *m* and *n* when injecting into inputs *i* and *j* when the delay is maximized and 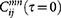 is the measured counts at minimum distinguishability as estimated from a Gaussian fit. The coincidence probability at minimal distinguishability depends on the relative phases of the elements *U_ij_*(*θ*). This leads to both constructive interference in which a coincidence peak is observed or destructive interference, seen as a coincidence dip, as the relative phase *θ* is tuned by the thermo-optic element. This effect is shown in Fig. 3(a), in which we see a coincidence dip at *θ* = 0 rad become a coincidence peak at *θ* = 0.94 rad. The measured visibilities after subtracting accidental coincidence counts for each pair of output ports when injecting |110〉 are shown in Fig. 3(b). These are compared with the visibilities predicted from the classical characterisation (shaded curves). Here we see agreement between the theory and experiment within error over most of the range of *θ*. Equivalent plots for the other two combinations of inputs are available in the supplement and show diminished agreement. This is attributed to the imbalance of losses between inputs 1 and 2 and input 3 reducing the accuracy of the numerical model used to calculate the predictions.

**Figure 4:**
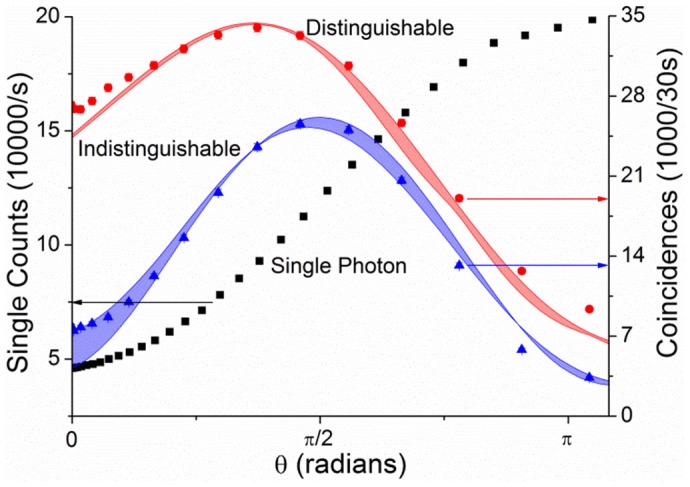
Measured single and coincidence counts as a function of phase. Black squares and left axis: single counts injecting into port 1 and measuring at port 1. Red and right axis: measured (circles) and predicted (band) coincidence counts at outputs 2 and 3 when injecting into ports 1 and 2 at maximal distinguishability. Blue and right axis: measured (triangles) and predicted (band) coincidence counts at outputs 2 and 3 when injecting into ports 1 and 2 at minimal distinguishability.

Two-photon non-classical fringes are obtained from the data described above by taking the coincidence counts at the point of minimal distinguishability. The resulting coincidence counts at outputs 2 and 3 when injecting into ports 1 and 2 are plotted in Fig. 4 (blue triangles) along with the theoretical prediction 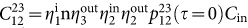. Here the relevant pairs of losses 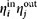 were solved for from the single photon measurements using the previously extracted unitary. These are overlayed with the single counts *N*_11_ (black squares) as well as the coincidences in the distinguishable case (red) for the same input and output ports, exhibiting a reduction in periodicity of both two-photon fringes compared with the single photon case. This is expected as there is a probability of two photons passing through the same interferometer arm in both the distinguishable and indistinguishable cases. However, the visibility of the two photon fringes is increased from 57% classically to 77% in the non-classical case due to the suppression of coincidences by quantum interference.

## Discussion

**Figure 5:**
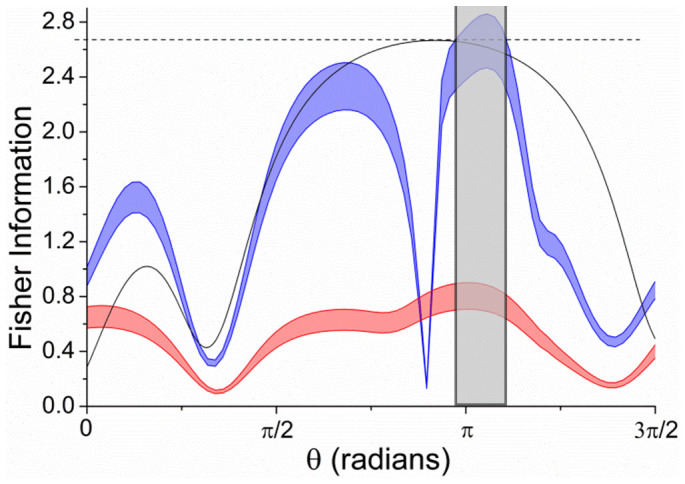
Extracted Fisher information of the three-arm interferometer when injecting |011〉 (blue curve) and single photons (red curve). Grey shaded region: values of the phase *θ* where the theoretical limit for an ideal three-path interferometer lies within the tolerance limit of the extracted Fisher information. The Fisher information calculated for an ideal, symmetric device with a two-photon input state is plotted (black curve) for comparison.

The analysis performed to this point now allows us to quantify the potential of our device for quantum metrology by extracting the classical Fisher information possible with our interferometer. Fisher information *F*(*θ*) is a measure of the amount of information that can be gained about an unknown measurand *θ* by sampling the measurement outcomes of a given probe system^1^. In this case, the measurand is the phase *θ* and the measurement outcomes are the number of photons present in each output mode. The Fisher information is then calculated by summing over all possible sets of nonclassical interference fringes 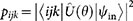 as follows:
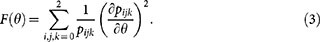
The Fisher information obtained from Eq. (3) with the |011〉 input state is shown in Fig. 5 (blue), surpassing the values obtainable with single photon inputs (red). The theoretical maximum of 8/3 ≈ 2.67 obtainable for photon pairs injected into an ideal three-arm interferometer also lies within the tolerance of the calculated *F*(*θ*) between 0.97*π* and 1.10*π* (grey shaded region). The sharp decrease seen at *θ* ≈ 0.89*π* is due to the fringes *p*_110_, *p*_011_, *p*_020_ and *p*_002_ all reaching an extremum at approximately the same point. Since this subset of fringes make the dominant contribution and the single fringe Fisher information for each is dependent on its first derivative, the result is a minimum in the total *F*(*θ*). Fisher information of *F* > *N*, where *N* is the number of photons in the probe state, is required to realize a quantum enhancement in an interferometric phase measurement^10^. Therefore, our result shows that phase measurements below the standard quantum limit should be possible with our device.

The variation of the Fisher information with the phase *θ* leads to a measurement precision that depends on the unknown phase. This, however may be counteracted by making use of an adaptive measurement scheme in which the device is tuned back to the value of *θ* corresponding to the optimal *F*(*θ*) using an adjustable feedback phase^12^. Such a scheme can be straightforwardly implemented in our device by using the demonstrated thermo-optic phase shifter as feedback, while the unknown phase would be imparted by another element such as a microfluidic channel^28^.

It is important to note that the phase *θ* in this device remained stable over the full duration of the experiment and that the measured visibilities were reproduced within the given errors despite the modest effort taken to stabilize it against temperature fluctuations and vibration. The stability and reproducibility of the phase supports the utility of our device for the adaptive measurement schemes described above. This contrasted with analogous fibre devices that have been reported to date, in which thermal fluctuations of the optical path of several wavelengths limited the study to a characterisation with a classical laser source^15^,^16^. The 3D fabrication capability of the FLDW technique may be further exploited to raise additional waveguides to the surface at points distributed over the length of the chip, allowing multiple interferometer arms to be phase-tuned in the way detailed in this paper. In this way, adaptive measurement of two unknown phases is possible^12^,^13^. This is a new and mostly unexplored type of measurement that is unique to multi-arm devices such as the one reported here. Furthermore this device could be used as a novel multiphoton state generator through integration with on-chip single photon sources^29^,^30^.

We have reported the first experimental demonstration of quantum interferometry in a 3D tunable laser-written three-port interferometer. The integrated geometry provided by FLDW enables improved stability compared to fibre and bulk set-ups, and the tunability illustrates the enhanced sensitivity achievable using non-classical states. The combination of high stability, 3D achitecture and tunability makes this device an ideal platform for practical and highly sensitive quantum sensing and an enabling technology for new multi-parameter estimation experiments.

## Methods

### Device fabrication

The interferometer was fabricated in alumino-borosilicate glass (Corning Eagle2000) by femtosecond laser direct-writing. This technique uses a femtosecond laser, focused inside a transparent substrate, to form localised refractive index change. The substrate can be translated with respect to the focus, thereby forming lines of index change which can act as waveguides^25^. The laser can be focused at multiple depths inside the sample meaning truly 3D waveguide circuits can be formed^31^, with high transmission^26^,^27^. In this study, the output of a Ti:Sapphire oscillator (Femtolasers GmbH, FEMTOSOURCE XL 500, 800 nm centre wavelength, 5.1 MHz repetition rate, <50 fs pulse duration) was focussed into the sample using a 100× oil immersion objective. The sample was translated with respect to the beam focus using Aerotech motion control stages with 10 nm precision. A pulse energy of 66 nJ and a sample translation speed of 1200 mm/min were selected with the sample being subjected to the rate annealing process described in^27^, yielding waveguides that were single mode at 800 nm. The waveguides were ellipsoidal in shape and supported modes with a diameter of approximately 6.5 *μ*m in the semi-major axis and 4.6 *μ*m in the semi-minor axis. A minimum coupling loss of 0.26 dB per facet was determined using a numerical calculation of the overlap integral between a measured waveguide and SM800 fibre mode. The waveguides follow a raised sine curve from an initial spacing of 127 *μ*m to a 7 *μ*m spacing in the 1.1 mm long interaction region over a distance of 8.175 mm. The four raised sine curves, two interaction regions and a straight section on the interferometer arms give a total device length of 40 mm. The curves have a minimum radius of curvature of 66 mm which has previously been shown to result in negligible bend loss^27^. Total insertion losses of 5.4 dB for input 1, 5.5 dB for input 2 and 12.5 dB for input 3 were measured by comparing the sum of the output intensities to the intensity launched into a single input. The measured insertion losses are much higher than the theoretical minimum due to small mismatches between the waveguide positions at the chip facets and those of the fibres in the commercial V-groove arrays used to couple into the chip which can deviate by up to 1 *μ*m from the optical axis in each direction. Drifts in alignment also occur during the curing process of the optical adhesive used to attach the V-grooves to the chip, further increasing the loss.

### Thermal tuning

The device was tuned thermo-optically using an 11 mm × 5.6 mm array of alumina thick film resistors with a resistance of 42 Ω mounted on the chip surface. The heater dissipated up to 7.6 W of power for an applied voltage of 18 V at the upper end of the tuning range and the I-V curve remained linear across the range of voltages considered in the experiment. A layer of thermally conductive compound was used to ensure uniform thermal contact between the heater and glass surface. The heater increased the temperature of the top surface while the bottom surface was held at a constant temperature of 20° C using a copper heatsink, leading to a linear temperature gradient across the thickness of the chip (see supplementary material).

### Photon pair generation

The fabricated three-arm interferometer was characterised using photons from a spontaneous parametric down-conversion (SPDC) source. The output of a continuous-wave, 402 nm laser diode (Toptica iBeam smart) was focussed into a 1 mm thick type-I phase matched BiBO crystal. Down converted photons were produced at 804 nm with a 6° opening cone angle and were passed through 3 nm bandpass filters before being coupled into polarization maintaining single mode fibres. One of the fibres was mounted on a computer controlled micron resolution actuator which translated one collection fibre with respect to another leading to a relative path delay. This was used to tune the temporal distinguishibility of the photons. The fibres were then coupled to the waveguides at the chip facets using commercial fibre V-groove arrays with a fibre spacing of 127 *μ*m matched to the waveguide spacing at the facets of the fabricated device. Photons were coupled out of the chip at the opposite facet by another V-groove array with standard single-mode fibres before being monitored by a set of silicon avalanche photodiodes (Excelitas).

## Supplementary Material

Supplementary Information Supplementary Information
