# Supplementary material for: Tunable quantum interference in a 3D integrated circuit
Source: Sci Rep. 2015 Apr 27;5:9601. doi: 10.1038/srep09601 (PMC5386201; doi:10.1038/srep09601)
Supplement: Supplementary Information [file srep09601-s1.pdf]

Tunable quantum interference in a 3D integrated circuit
-Supplementary Material-

Zachary Chaboyer,[1,*] Thomas Meany,[1] L. G. Helt,[1] Michael J. Withford,[1] and M. J. Steel[1]

[1]*Centre for Ultrahigh bandwidth Devices for Optical Systems (CUDOS), MQ Photonics Research Centre, Department of Physics and Astronomy, Macquarie University, NSW 2109, Australia*

Thermo-optic phase shifter

The implemented tunable interferometer uses a resistive heater mounted on the chip surface to induce a steady state temperature gradient within the chip, elevating the temperature of the raised arm with respect to the other two, leading to a relative phase shift due to the thermo-optic effect. Our combination of laser writing parameters and the use of an oil-immersion objective did not allow waveguides to be written close enough to the surface to be heated independently. Instead, the heating element increases the temperature of the top surface over an area that is much larger than the dimensions of the interferometer. This both makes the system robust to alignment with the target waveguide and means that the temperature gradient has no lateral dependence within the vicinity of the device. The temperature of the raised interferometer arm is therefore raised by an equal amount with respect to each of the buried arms. We model the temperature gradient within the heater and chip as the solution to the steady state 1D heat equation within the domain $0 \leq z \leq L = L_h + L_c$

$$-\kappa(z)\frac{d^2T}{dz^2} = Q(z). \tag{S1}$$

Here $L_h = 1.1$ mm and $L_c = 1.1$ mm are the thicknesses of the heater and chip, respectively, and $Q(z)$ is the volume power density of the heater, having a constant value for $0 \leq z \leq L_h$, and zero elsewhere. The thermal conductivity $\kappa(z)$ is set to the value of 30 W/mK for alumina within the heater and 0.9393 W/mK as quoted by the manufacturer for Eagle2000 within the chip. The power density is calculated by dividing the dissipated electrical power IV by the total volume of the heating element. The end of the domain is held at a constant temperature of 20° C to reflect the action of the heat sink at the bottom surface. Since conductive heat transfer into the system dominates at the top face of the chip, an insulating boundary condition $dT/dz = 0$ is enforced at $z = 0$. The temperature distribution obtained after solving Eq. S1 is plotted in Fig. S1.

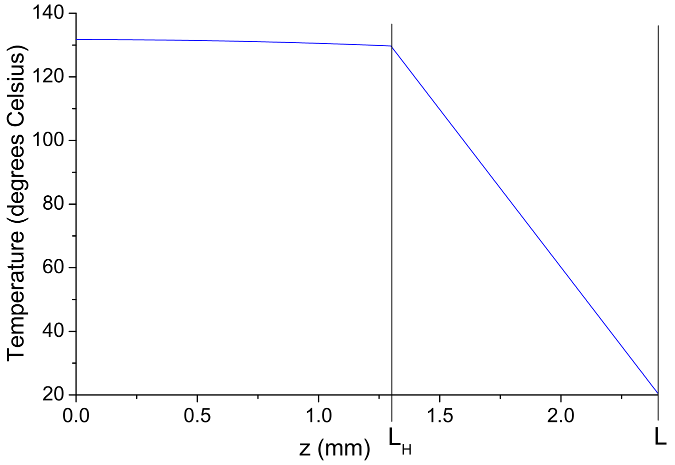

FIG. S1: Calculated 1D temperature distribution for a dissipated power of 5.1 W. The temperature gradient becomes linear within the chip (1.1 mm$\leq z \leq$ 2.2 mm).

---

*Electronic address: `zachary.chaboyer@students.mq.edu.au`

Fig. S1 shows that the temperature distribution becomes linear for $L_h \leq z \leq L$. Given a typical value of $dn/dT \approx 3 \times 10^{-6}$ for the thermo-optic coefficient in borosilicate glass [S1], a temperature difference of approximately 13° C between the raised and buried waveguides is required for light propagating in the raised arm to acquire a relative phase of π over the length of the heater. This translates to a 115° C difference between the top and bottom surfaces. This matches extremely well with the calculated gradient of 109° C between $z = L_h$ and $z = L$ in Fig. S1.

Extraction of phase-dependent unitary

Our three-arm interferometer may be represented by a 3×3 unitary transformation exhibiting a dependence on the relative phase induced in the middle arm by the thermo-optic phase shifter. Here we detail the extraction of this unitary as a function of the induced phase from a set of measured classical interference fringes. For this characterisation, we use photons from a single collection arm of a photon pair source based on spontaneous parametric downconversion in a nonlinear crystal. Since classical interference is essentially a single photon phenomenon, the fringes obtained in this way are equivalent to those that would be obtained using classical bright light. Photons were injected into each input port, while single counts were monitored at each output using silicon avalanche photodiodes. The three sets of single photon counts as a function of the voltage applied to the resistive heater on the chip surface are shown in Fig. S2.

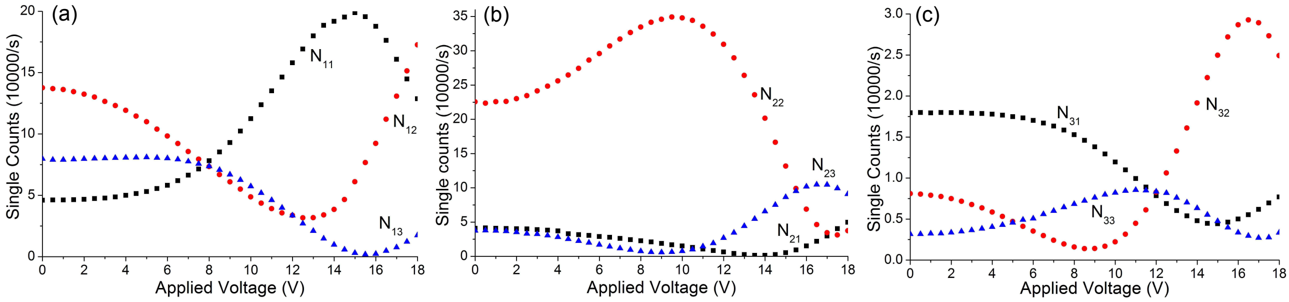

FIG. S2: Counts at each output port as a function of voltage applied to the resistive heater when injecting single photons into (a) input 1, (b) input 2, (c) input 3. The error bars are smaller than the data points and are omitted.

The unitary matrix describing the device consists of both real transition amplitudes and complex phases [S2]. The method described in [S2] allows the transition amplitudes of an arbitrary N port device to be determined from the N^2 possible intensity (or single photon count) measurements, while it is possible to solve for the phases using measured two-photon visibilities. However, if we wish to predict two-photon data using only single-photon measurements, we must reduce the number of free parameters. This is done by considering our interferometer as a three-port device characterised by a coupling matrix C that varies with the induced phase θ in the middle arm. The coupling matrix describes the evolution of the input modes $\hat{\boldsymbol{a}} = (\hat{a}_1, \hat{a}_2, \hat{a}_3)$ to the output modes $\hat{\boldsymbol{b}} = (\hat{b}_1, \hat{b}_2, \hat{b}_3)$ along the propagation direction y according to

$$\frac{d\hat{b}}{dy} = -iC(y,\theta)\hat{a}, \tag{S2}$$

where

$$C(y,\theta) = \begin{pmatrix} \beta & G_1(y,\theta) & G_3(y,\theta) \\ G_1(y,\theta) & \beta & G_2(y,\theta) \\ G_3(y,\theta) & G_2(y,\theta) & \beta \end{pmatrix}. \tag{S3}$$

We solve Eq. (S2) approximately by considering an effective coupling matrix $\tilde{C}(\theta)$ taking the same form as (S3), with effective coupling coefficients $\bar{G}_i$ (see Fig. S3(a)). This gives the approximate solution $\hat{b} = e^{-i\tilde{C}}\hat{a}$, allowing the unitary transformation $U(\theta)$ governing the evolution of the device's input to output modes to be determined as the matrix exponential of the effective coupling matrix. The counts N_{ij} at each output port j when injecting M number of photons into port i are related to this transfomation by

$$N_{ij} = \eta_i^{\mathrm{in}} \eta_j^{\mathrm{out}} \left| U_{ij} \right|^2 M \tag{S4}$$

The input and output losses η_i^{in} and η_j^{out} (in which the output loss includes both the facet loss and the non-unit efficiency of the detector monitoring output j) are accounted for by forming appropriate ratios of single counts for

which the losses cancel [S2]

$$F_{ijkl} = \frac{N_{ij}N_{kl}}{N_{il}N_{kj}}. \quad \text{(S5)}$$

We now fit the theoretically determined ratios F_{1122}, F_{1133} and F_{2233} to their measured values at each value of θ using a maximum likelihood technique [S3], assuming that the ratios follow a Gaussian distribution. The log-likelihood function then takes the following form weighted against the experimental errors σ_{ijkl}

$$L(V) = \sum \frac{(F_{ijkl} - \frac{|U_{ij}|^2|U_{kl}|^2}{|U_{il}|^2|U_{kj}|^2})^2}{2(\sigma_{ijkl})^2}. \quad \text{(S6)}$$

The function L takes the form of a Gaussian near the converged values of the coupling coefficients when plotted as a function of each G_i while holding the others constant. This allows tolerances for each to be determined based on the $1/e^2$ width of each distribution. The resulting upper and lower bounds for each coupling coefficient G_i are plotted in Fig. S3(b) as shaded bands. A value of $U(V)$ is determined at each data point using a routine that iterates through a set of nine arrays of single count measurements, minimizing $L(V)$ at each data point using a simplex algorithm. This yields a set of coupling coefficients as a function of the electrical power $P = IV$ dissipated by the heater. We can then determine the induced phase by fitting the power-dependent data to the expression $|U_{ij}(\theta)|^2 = A\sin(kIV) + B\cos(kIV)$. The obtained $|U_{ij}|^2$ are plotted in Fig. S4 and are equivalent to a normalisation of the measured fringes accounting for the different facet losses at each input and output port.

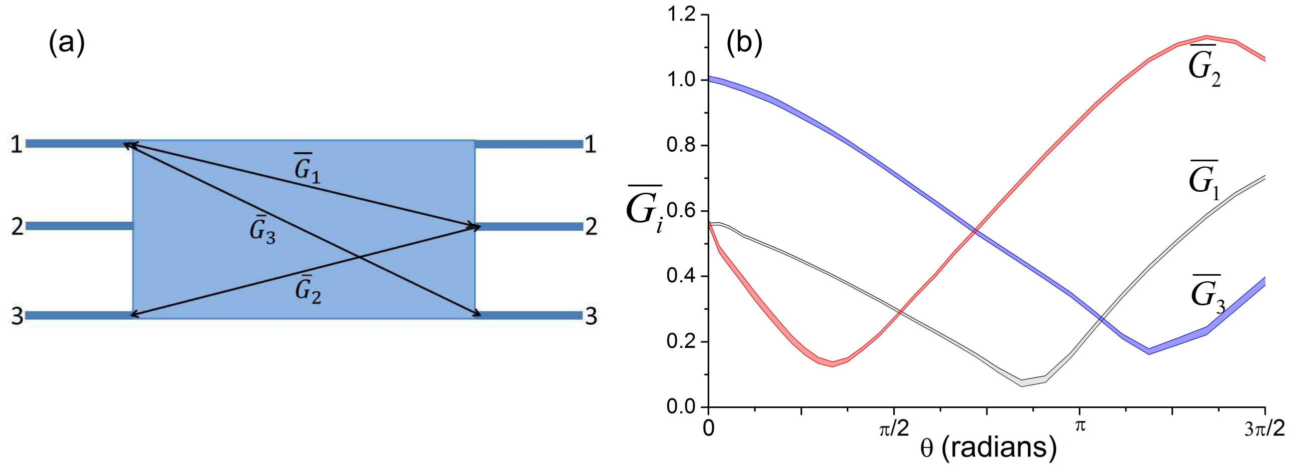

FIG. S3: (a) Schematic of the three-port device described by effective couplings G_1, G_2 and G_3. (b) Tolerance limits (represented by shaded bands) of the extracted coupling coefficients plotted as a function of induced phase in the middle interferometer arm.

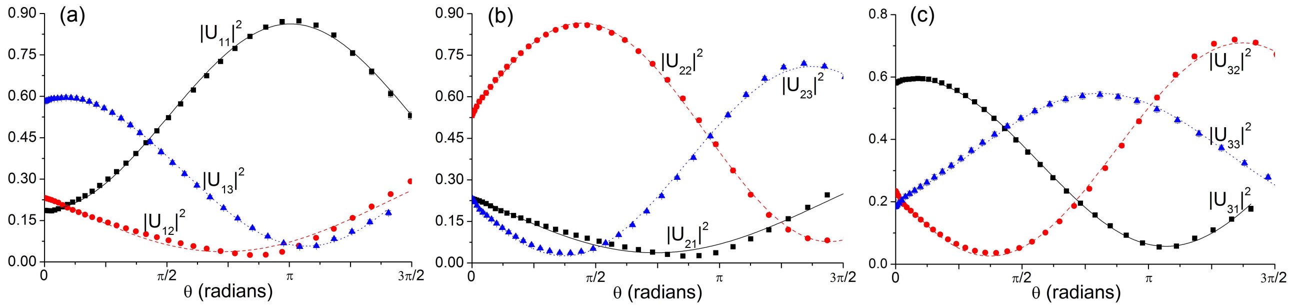

FIG. S4: Extracted $|U_{ij}|^2$ as a function of induced phase for (a) $i = 1$, (b) $i = 2$, (c) $i = 3$.

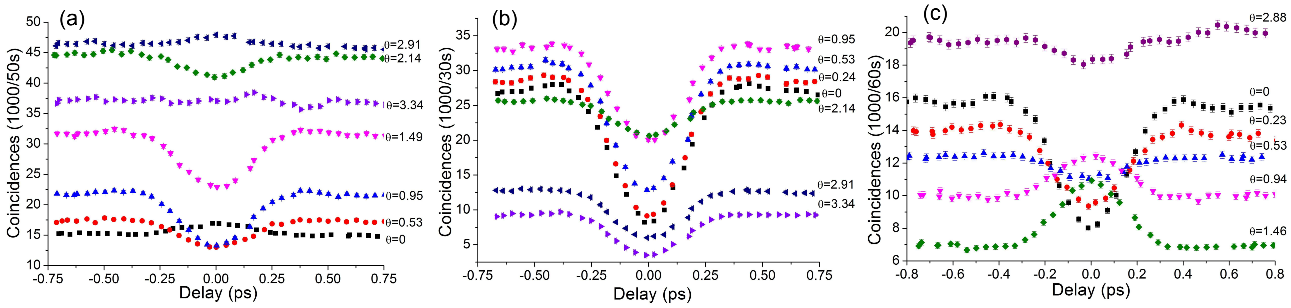

FIG. S5: Two-photon coincidences as a function of relative delay at various values of heater voltage while injecting $|110\rangle$ and (a) measuring $|110\rangle$, (b) measuring $|011\rangle$, (c) measuring $|101\rangle$.

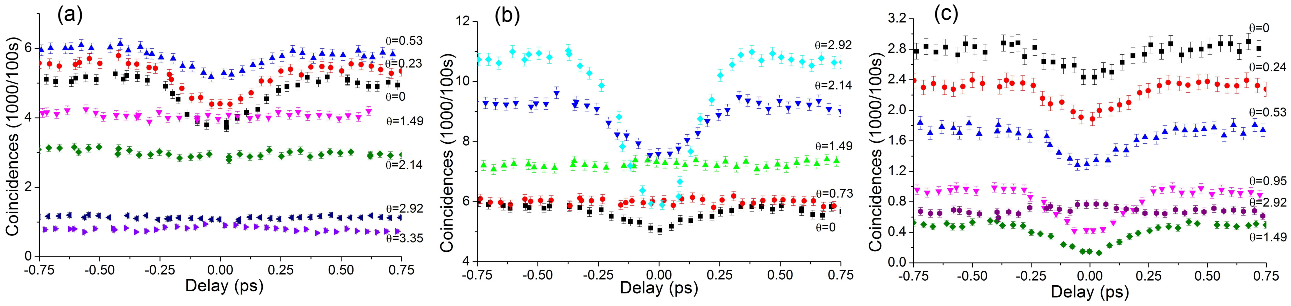

FIG. S6: Two-photon coincidences as a function of relative delay at various values of heater voltage while injecting $|011\rangle$ and (a) measuring $|110\rangle$, (b) measuring $|011\rangle$, (c) measuring $|101\rangle$.

Quantum Characterisation

A quantum characterisation of the device was performed by injecting 804 nm photon pairs into each combination of input modes while measuring coincidences at each combination of outputs. We control the distinguishability of the photons by means of a temporal delay varied using a servo motor that translates the free-space to fibre coupling mount. A full scan of the temporal delay between the photons was performed at each value of the induced phase. Control of the two-photon interference by means of the phase shifter is evidenced by the observed change in the visibility and the transition from interference dips to peaks (Figs. S5-S7). The visibility of each two-photon scan was determined by fitting each set of coincidence counts to a Gaussian and then comparing the counts at maximal delay $C_{mn}(\tau_{\max})$ (the baseline) to those at minimal delay $C_{mn}(\tau = 0)$ (corresponding to the extrema of the Gaussian fit)

$$V_{ij}^{mn} = \frac{C_{mn}(\tau_{\max}) - C_{mn}(0)}{C_{mn}(\tau_{\max})} \tag{S7}$$

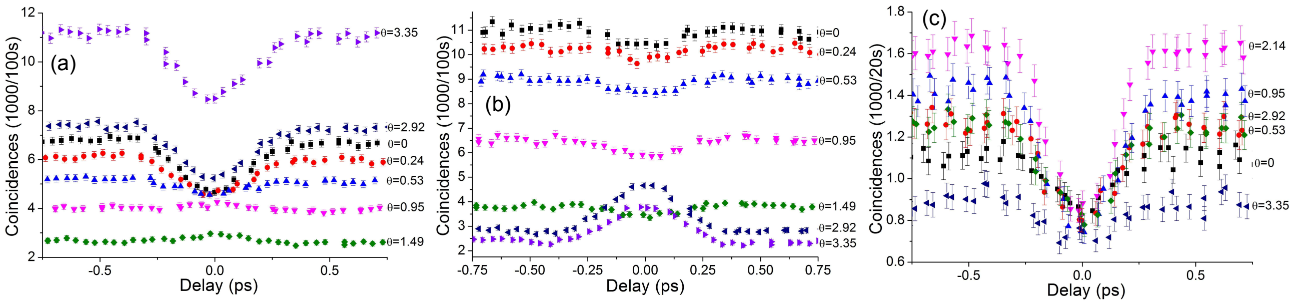

FIG. S7: Two-fold coincidences as a function of relative delay at various values of heater voltage while injecting $|101\rangle$ and (a) measuring $|110\rangle$, (b) measuring $|011\rangle$, (c) measuring $|101\rangle$.

where a negative value corresponds to a coincidence peak and a positive value denotes a coincidence dip. The transition from the coalescence effect of reduced coincidences to an enhancement of coincidences with the induced phase can be understood mathematically by considering a two-photon state being injected into an arbitrary optical multiport network. When two otherwise indistinguishable photons are incident at inputs i and j of a network described by a unitary matrix U, we find that the probability of finding photons at output ports m and n is

$$p_{ij}^{mn}(\tau) = |U_{im}U_{jn}|^2 + |U_{in}U_{jm}|^2 + 2\mathrm{Re}(U_{im}U_{jn}U_{jm}^*U_{in}^*)e^{-\kappa\tau^2/2} \tag{S8}$$

where κ is a constant related to the frequency distribution of the photons. We see that this approaches the classical value as the delay becomes infinitely large, while the quantum interference term (third) becomes important as the delay approaches zero, corresponding to the case of minimum distinguishability. It can also be seen that the interference term can take a positive or negative value depending on the complex phase dependence of the matrix elements U_{ij}, allowing for either destructive interference resulting in a coincidence dip or constructive interference leading to a coincidence peak. The visibility of the measured quantum interference can be predicted using the extracted unitary according to the definition above

$$\begin{aligned} V_{ij}^{mn} &= \frac{p_{ij}^{mn}(\tau_{\max}) - p_{ij}^{mn}(\tau = 0)}{p_{ij}^{mn}(\tau_{\max})} \\ &= -\frac{2\mathrm{Re}(U_{im}U_{jn}U_{jm}^*U_{in}^*)}{|U_{im}U_{jn}|^2 + |U_{in}U_{jm}|^2} \end{aligned} \tag{S9}$$

The predicted and measured visibilities for each combination of input and output ports are plotted in Fig. S8. Note that the agreement between experiment and theory is worse in Figs. S8 (b) and (c). We attribute this to the larger loss for input 3 causing the ratios F_{1133} and F_{2233} to approach very large or small values, reducing the accuracy of the numerical model used to extract the unitary.

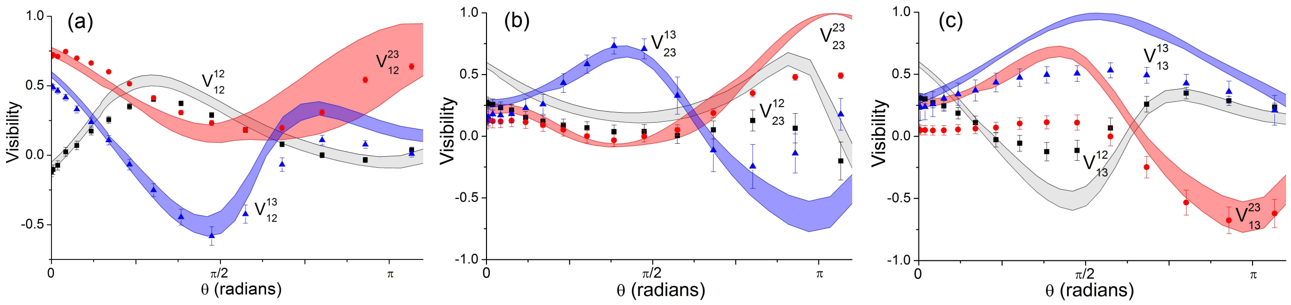

FIG. S8: Visibilities as a function of induced phase when (a) injecting $|110\rangle$, (b) injecting $|011\rangle$, (c) injecting $|101\rangle$. Black: measuring $|110\rangle$, red: measuring $|011\rangle$, blue: measuring $|101\rangle$. Points: measured values determined from a Gaussian fit. The larger experimental error bars in b) and c) are due to the imbalance of insertion losses for inputs 1 and 2 compared to input 3 reducing the signal-to-noise ratio when injecting $|011\rangle$ and $|101\rangle$. Curves: predictions based on the unitary extracted from the classical characterisation.

Extracted Fisher Information

The Fisher information quantifies the information that can be gained about a measurand θ from a given probe system by sampling its possible measurement outcomes x. It is obtained from the probabilities $p(x|\theta)$ of measuring x given a particular value of θ through a summation over all the possible outcomes

$$F(\theta) = \sum_j \frac{1}{p(x_j|\theta)} \left(\frac{dp(x_j|\theta)}{d\theta} \right)^2 \tag{S10}$$

In our case, the probe system is the three-arm interferometer, the measurand is an unknown phase and the measurement outcomes are the number of photons present in each output mode. We may predict the Fisher information achievable with our device by summing over all possible sets of nonclassical interference fringes $\langle\psi_{\mathrm{out}}| U(\theta) |\psi_{\mathrm{in}}\rangle$. These

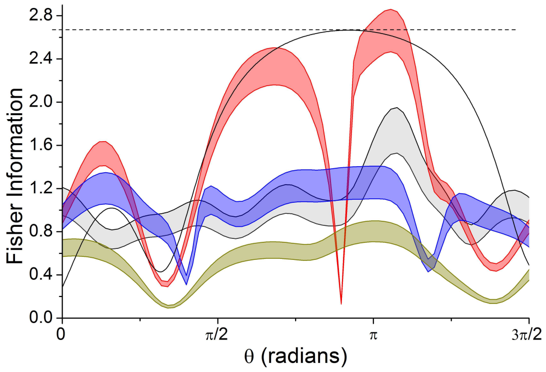

FIG. S9: Fisher information for two-photon input states calculated from the extracted unitary. Grey band: injecting $|110\rangle$, red band: injecting $|011\rangle$, blue band: injecting $|101\rangle$, yellow band: single photon input, black curve: calculated for $|110\rangle$ injected into an ideal device.

are calculated taking the input state obtained by applying creation operators $\hat{a}_i$ to the vacuum in input state i, and mapping these to the output operators $\hat{b}_i$ according to the unitary transformation U determined above [S4]

$$\hat{a}_i\hat{a}_j |0\rangle \rightarrow \hat{b}_i\hat{b}_j |0\rangle = (\sum_{m=1}^{3} U_{im}\hat{a}_m)(\sum_{n=1}^{3} U_{jn}\hat{a}_n) |0\rangle \tag{S11}$$

After expanding and simplifying, we take the inner product with a given output state $\langle\psi_{\text{out}}| = \langle 0| \hat{a}_m^\dagger \hat{a}_n^\dagger$. Since the terms with $m, n \neq i, j$ disappear, the probability is given by the coefficient of each term $\hat{a}_i\hat{a}_j$. The probability p_{ij}^{mn} of finding photons at outputs m and n when exciting distinct inputs i and j now takes the form

$$p_{ij}^{mn} = \frac{1}{1+\delta_{mn}} |U_{im}U_{jn} + U_{in}U_{jm}|^2. \tag{S12}$$

In the case of $m \neq n$ (S12) gives the probability of measuring a coincidence at outputs m and n, while $m = n$ corresponds to two photons emerging from output m. Since the transformation U was not determined in a closed form, each nonclassical fringe must again be calculated "point-by-point", first finding $U(\theta) = e^{-i\tilde{C}(\theta)}$ at each value of θ. The Fisher information is then calculated according to (S10), with the derivatives of each curve being calculated numerically. The calculated Fisher information for each possible two-photon input state $|110\rangle$, $|011\rangle$ and $|101\rangle$ is shown in Fig. S9.

[S1] Jewell, J. M. Thermooptic coefficients of some standard reference material glasses. *Journal of the American Ceramic Society* **74**, 1689–1691 (1991).

[S2] Laing, A. & O'Brien, J. Super-stable tomography of any linear optical device. *arXiv preprint* (2012). 1208.2868v1.

[S3] Meany, T. *et al.* Non-classical interference in integrated 3D multiports. *Optics Express* **20**, 26895–26905 (2012).

[S4] Brougham, T., Košták, V., Jex, I., Andersson, E. & Kiss, T. Entanglement preparation using symmetric multiports. *The European Physical Journal D* **61**, 231–236 (2010).
